# Comparative assessment of *Cucurbita moschata* seed polypeptides toward the protection of human skin cells against oxidative stress-induced aging

**DOI:** 10.3389/fnut.2022.1091499

**Published:** 2023-01-04

**Authors:** Chunhuan Liu, Peiyu Wang, Cheng Yang, Bingtian Zhao, Peidong Sun

**Affiliations:** Key Laboratory of Food Colloids and Biotechnology, Ministry of Education, School of Chemical and Material Engineering, Jiangnan University, Wuxi, China

**Keywords:** *Cucurbita moschata* seed polypeptides, cell growth, oxidative stress, skin anti-aging, human skin fibroblasts

## Abstract

Skin aging has attracted much attention among the current aging population of society. The seeds of *Cucurbita moschata* possess a variety of potential biological activities as a healthy diet. However, limited information is available on the skin-antiaging properties of *C. moschata* seed protein and its hydrolysate. Herein, we developed a novel strategy for protecting human skin cells against oxidative stress-induced aging by *C. moschata* seed polypeptides. *C. moschata* seed polypeptides (*CSPs*) with different molecular weight distributions were successfully prepared by controlling the protease hydrolysis time. The proportions of < 1,000 Da polypeptides of P-1, P-2, and P-3 were 0.11, 20.26, and 92.72%, respectively. P-3 contained the highest proportion of polypeptides of size < 1,000 Da, which was observed to promote human skin fibroblast (HSF) growth by MTT assay, cell cycle, and morphology. P-3 has an efficient repair effect on the H_2_O_2_-induced aging of HSF cells. To explain this phenomenon, cell lifespan, intracellular ROS level, superoxide dismutase (SOD) activity, and glutathione (GSH) content were investigated to reveal the interactions between P-3 and antiaging. With the increase in P-3 concentration, the ROS level significantly decreased, and the SOD activity and GSH content significantly increased in H_2_O_2_-induced HSF cells. These findings indicated that *CSPs* have the potential to inhibit skin aging, which could be advantageous in the health industry for providing personal care.

## 1. Introduction

With the emergence of an aging population and the gradual deepening of health consciousness in the minds of the current society, skin aging has attracted much attention (1). Skin aging manifests as wrinkles, loose skin, and decreased elasticity. From a microscopic perspective, skin aging is interpreted as cell senescence (2). Specifically, the number of human skin fibroblast (HSF) cells located in the dermal layer of the skin reduces, the cell growth stops, and the secretion of collagen and elastin reduces, thereby resulting in the formation of wrinkles. Therefore, maintaining the population of HSF cells is important both for preventing and for treating age-related skin changes.

Oxidative stress is considered an important mechanism of skin aging as it triggers the apoptosis of various cell types, especially HSF cells, which plays an important role in maintaining healthy young skin (3). It reduces the number of HSF cells by inducing apoptosis and decreasing their regenerative capacity, leading to increased skin sagging. Therefore, the suppression of oxidative stress-induced apoptosis of HSF cells is a prevention strategy for maintaining healthy, youthful skin. Currently, materials that can effectively inhibit oxidative stress-induced apoptosis include flavonoids (4, 5), polyphenols (6, 7), and so on. However, these materials are difficult to prepare and expensive, which limits their application in industry. Therefore, it is urgent to find materials that not only could inhibit oxidative stress-induced apoptosis but also are natural and accessible (8).

Protein-derived products, peptides, and protein hydrolysates from natural sources were reported to have a skin-lightening effect and are used to treat skin diseases (9–11). Enzymatic hydrolysis is a highly efficient and environment-friendly approach to cleave proteins to release biologically active peptide fractions (12–16). The polypeptides prepared by protease hydrolysis are mainly from animal and plant proteins (17). The polypeptides prepared by the hydrolysis of animal proteins have various functions, but they also have many disadvantages, such as unpleasant smell, high bacterial risk, and high price (18). The polypeptides from plant proteins have attracted a great deal of attention recently as they are relatively cheap, natural, safe, and abundant (19). In addition, the peptides prepared by hydrolyzing plant proteins showed good ROS scavenging ability (20, 21). Therefore, polypeptides from plants have been considered promising antiaging candidates. Unfortunately, most studies are focused on the separation and purification of polypeptides with high antioxidant properties and the identification of their structures. Furthermore, the determination of antioxidant performance is mainly based on *in vitro* chemical methods which cannot evaluate the bioactivity of polypeptides, especially the bioactivity of human cells. The relationship between the molecular weights of polypeptides and bioactivity is unclear (22, 23). Therefore, it is essential to investigate the effects of plant polypeptides with different molecular weights on the antioxidant properties of human cells, e.g., oxidative stress-induced aging.

*Cucurbita moschata* seed (CS) is an important industrial crop. It has been used as a protein-rich food ingredient and nutritional health agent in many countries for thousands of years (24, 25). CS is recognized as an excellent medicinal and edible plant seed that helps reduce hypertension and treat prostate hyperplasia and diabetes (26–28). In our previous study, CS protein was found to have a high-quality protein containing beneficial amino acid composition. Thereby, CS protein showed the potential to be investigated as an alternative ingredient in cosmetic and nutraceutical industries. Unfortunately, there is limited literature on skin antiaging by using *C. moschata* seed polypeptides (CSPs).

Herein, a novel strategy to protect HSF cells against oxidative stress-induced aging by the CSP was developed. First, polypeptides with different molecular weight distributions were obtained by tuning the time of protease hydrolysis. Subsequently, we established an H_2_O_2_-induced oxidatively damaged HSF cell model to screen oxidative stress inhibitory CSP. Finally, the effects of CSP on the cell population doubling level (PDL), the intracellular ROS level, intracellular superoxide dismutase (SOD), glutathione (GSH), and senescence-associated β-galactosidase staining were systematically investigated. This study attempted to understand the effects of CSP on protecting HSF cells from oxidative stress-induced aging and revealed its contribution to skin antiaging.

## 2. Materials and methods

### 2.1. Materials

Defatted *C. moschata* seed meal was obtained from Anyang Jingsen Biotechnology Co., Ltd. (Henan, China). Alcalase (200 U/mg) and trypsin (250 U/mg) were obtained from Solarbio (Beijing, China). Dulbecco's Modified Eagle Medium (DMEM), streptomycin, and fetal bovine serum (FBS) were obtained from BNCC (Beijing, China). Hydrogen peroxide (H_2_O_2_) and 3-(4,5-dimethylthiazol-2-yl)-2,5-diphenyltetrazolium bromide (MTT) were purchased from Sigma Biochemicals (St. Louis, MO, USA). 2′,7′-dichlorodihydrofluorescein diacetate (DCFH-DA), superoxide dismutase (SOD) assay kit, and GSH assay kit were purchased from Beyotime (Nanjing, China). Other chemicals used in the study were of analytical grade.

### 2.2. Extraction of *Cucurbita moschata* seed protein

*CS protein* was extracted from defatted *C. moschata* seed meal and prepared according to the method of Wang et al. (29) with slight modifications. Briefly, defatted *CS* obtained after air-drying overnight at room temperature was added to deionized water in a ratio of 1:30 (w/v). Then, the pH was adjusted to 9.5 using 0.5 M NaOH. After extraction in an ultrasonic bath (KQ-300DE, Kunshan Ultrasonic Instruments Co., Ltd., China) for 3 h at room temperature, the samples were centrifuged at 5,000 × g for 20 min to prepare the supernatant. The supernatant was adjusted to pH = 4.3 with 1 M HCl and then centrifuged at 10,000 g for 20 min. Finally, the precipitation was washed with distilled water and freeze-dried at −30°C at 39 Pa for 72 h by using a vacuum freeze dryer (10N/C, Scientz, China) to obtain the *CS* protein powder.

### 2.3. Preparation of CSP with different molecular weights

*CS* protein powder was hydrolyzed by both Alcalase and trypsin at different time periods (as shown in Supporting information Supplementary Table S1) to obtain P-1, P-2, and P-3, respectively. *CS* protein powder was dispersed in distilled water to obtain a 0.05 mg/ml dispersion. Then, the dispersion was incubated with Alcalase (0.025 mg/ml, enzyme/substrate) at pH 9.0 and 50°C for 10 min and 120 min for P-2 and P-3, respectively. Later, the obtained dispersion was immersed in boiling water for 10 min to inactivate the enzyme. After cooling down to room temperature, the obtained dispersion was incubated with trypsin (0.025 mg/ml, enzyme/substrate) at pH 7.5 and 37°C for 10 min and 120 min to obtain P-2 and P-3, respectively. The control (P-1) was prepared under the same incubation conditions but without enzyme addition. The obtained dispersion was immersed in boiling water for 10 min to inactivate the enzyme and then cooled down to room temperature and adjusted to a neutral pH. Finally, the dispersion was centrifuged at 5,000 × g for 20 min and then freeze-dried at −30°C at 39 Pa for 72 h using a vacuum freeze dryer (10N/C, Scientz, China) to obtain the *CSP* powder. The amount of enzyme/substrate ratio is based on the freeze-dried sample prepared as described in the “Extraction of Cucurbita moschata seed protein” section. The CSP powder was then stored in a refrigerator at −20°C.

### 2.4. Determination of the molecular weight distribution of CSP

The molecular weight distribution of CSP was determined by using a dextran gel column [Waters 1525EF with TSK gel 2000 SW_XL_ (300 × 7.8 mm)] equipped with a UV 220 nm detector. Glycine-Glycine-Glycine (MW189), Glycine-Glycine-Tyrosine-Arginine (MW451), Bacitracin (MW1450), and Cytochrome C (MW12400) were used as standard compounds for calibrating the molecular weights of the samples. The injection volume was 20 μl of the 10-mg/ml sample in ultrapure water. The mobile phase consisted of acetonitrile, water, and trifluoroacetic acid (40:60:0.1, v/v), and its flow rate was 0.5 ml/min.

### 2.5. Human skin fibroblast cell cultures and cell viability assay

Human skin fibroblasts (Normal Human Skin Fibrotic Cell Line (HSF), BNCC353686) were purchased from BNCC (Beijing, China). HSF cells were cultured in DMEM containing 10% FBS and 1% penicillin-streptomycin and grown in a 5% CO_2_ incubator set at 37°C. The tested cell lines were observed with an inverted microscope (OLYMPUS CKX41) and photographs were taken.

The effects of CSP on the viability of HSF cells were investigated by 3-(4,5-dimethylthiazol-2-yl)-2 and 5-diphenyltetrazolium bromide (MTT, Sigma Aldrich) assay. The HSF cells were seeded in 96-well plates at 2 × 10^5^ cells/ml and immediately placed in an incubator for 24 h. DMEM was used as a solvent to prepare P-1, P-2, and P-3 solutions of concentrations 0.2–1.0 mg/ml with 0.2 mg/ml increments. Then, the sample solutions were added to the well and incubated with cells for 24 h. After that, MTT was added to the plates and incubated for another 4 h at 37°C in a 5% CO_2_ incubator. Finally, DMSO was added to the 96-well plates, and the absorbance of each well at 490 nm was tested using a Varioskan LUX (Thermo Fisher). The cell viability was examined by MTT assay and reported as percent viability compared to the control wells (containing cells without any peptide treatments). All evaluated samples were conducted under non-toxic conditions against cell lines.

### 2.6. Cell cycle assay

Human skin fibroblast cells were seeded in 6-well plates at 2 × 10^5^ cells/ml and placed in an incubator for 24 h. DMEM was used as a solvent to prepare P-1, P-2, and P-3 solutions with different concentrations, and then the sample solutions were added to the well and incubated with cells for 24 h. After that, we collected the cells by following the steps in the cell cycle kit. Briefly, the cells were collected and washed with PBS buffer two times. Then, the cells were resuspended in 500 μl of binding buffer (containing 1% propidium iodide) and incubated for 30 min at 37°C in the dark. A flow cytometer (BD FACSArica III) equipped with three lasers, namely, 405 nm, 488 nm, and 633 nm was used for detection. The ModFit analysis was used based on the obtained data.

### 2.7. Measurements of CSP on repair effects of HSF cells by H_2_O_2_ stress

#### 2.7.1 Cell culture and cell viability

First, we established an oxidative stress model by treating the HSF cells with different concentrations (1.0, 1.2, 1.4, and 1.6 mM) of H_2_O_2_ and for different times (1, 2, 3, and 4 h). HSF cells were seeded in a 96-well plate, and after 24 h of incubation, the solutions in the wells were removed. To determine the appropriate H_2_O_2_ concentrations and treating times, different molar concentrations of H_2_O_2_ solutions were prepared with DMEM as the solution. Notably, 100 μl of the sample solution was added per well for 1 h, and 100 μl of DMEM was used instead of the sample as the control group. As shown in Supplementary Figure S1, HSF cells treated with 1.4 mM H_2_O_2_ for 1 h were chosen for the subsequent oxidative damaging experiment. After establishing this model, the cell viability of HSF cells after incubation with 0.6 mg/ml CSP for 24 h was examined. The cell viability was determined according to the “Human skin fibroblasts (HSF) cell cultures and cell viability assay” section.

#### 2.7.2 Morphology

HSF cells were seeded in 6-well plates at 2 × 10^5^ cells/ml according to the above methods. The morphology of the HSF cells was observed using an inverted microscope.

#### 2.7.3 Cell population doubling level

PDL refers to the multiplication times of the cell group from the beginning of culture till measurement, which could directly reflect the life span of cells. The living cells were stained with a trypan blue stain solution. The total number of cells was recorded every time the cells were passaged. During the culture, fresh P-3 solution was added continuously.

#### 2.7.4 Intracellular ROS measurements

Intracellular ROS were measured using a DCFH-DA fluorescent probe kit. H_2_O_2_-induced HSF cells were treated with 0.6 mg/ml P-3 for 24 h. The HSF cells only treated with DMEM for 24 h were used as the blank. The control group was treated with 1.4 mM H_2_O_2_ for 1 h. HSF cells were seeded in black 96-well microplates at 20 × 10^3^ cells/ml. The cells were labeled with DCFH-DA (10 μM/L) for 30 min in the dark. At the end of the incubation time, the cells were washed with PBS water (2 × ). Then, Varioskan LUX (Thermo Fisher) and a laser confocal microscope (Leica SD) were used to detect the fluorescence intensity of the HSF cells.

#### 2.7.5 Intracellular SOD and GSH measurements

SOD could catalyze the superoxide anion disproportionation reaction and generate H_2_O_2_ and a molecule of oxygen. GSH is a tri-peptide that is composed of glutamic acid, glycine, and cysteine, and it is an intracellular free radical scavenger. Both SOD and GSH can remove intracellular free radicals, and hence, they can play an antiaging role. The SOD and GSH contents of the cells were measured according to the instructions in the kit.

#### 2.7.6 Senescence-associated β-galactosidase staining

Cell senescence-associated β-galactosidase is used for identifying senescent cells based on the increase in senescence-related β-galactosidase activity during cell senescence. The kit used 5-bromo-4-chloro-3-indolyl β-D-galactopyranoside (X-GAL) as a substrate and produced a dark blue product under the catalysis of aging-specific β-galactosidase. Finally, we observed the staining using an optical microscope.

### 2.8. Statistical analysis

Data were expressed as means ± the standard error of the mean, and the results were derived from at least three independent experiments. All data were analyzed by Tukey's multiple comparison *post-hoc* test using the GraphPad Prism software (version 7). ^**^ and ^***^ indicate the differences in *p-*values of < 0.05 and < 0.001, respectively.

## 3. Results and discussion

### 3.1. Preparation of CSP with different molecular weight distributions

**Figure 1:**
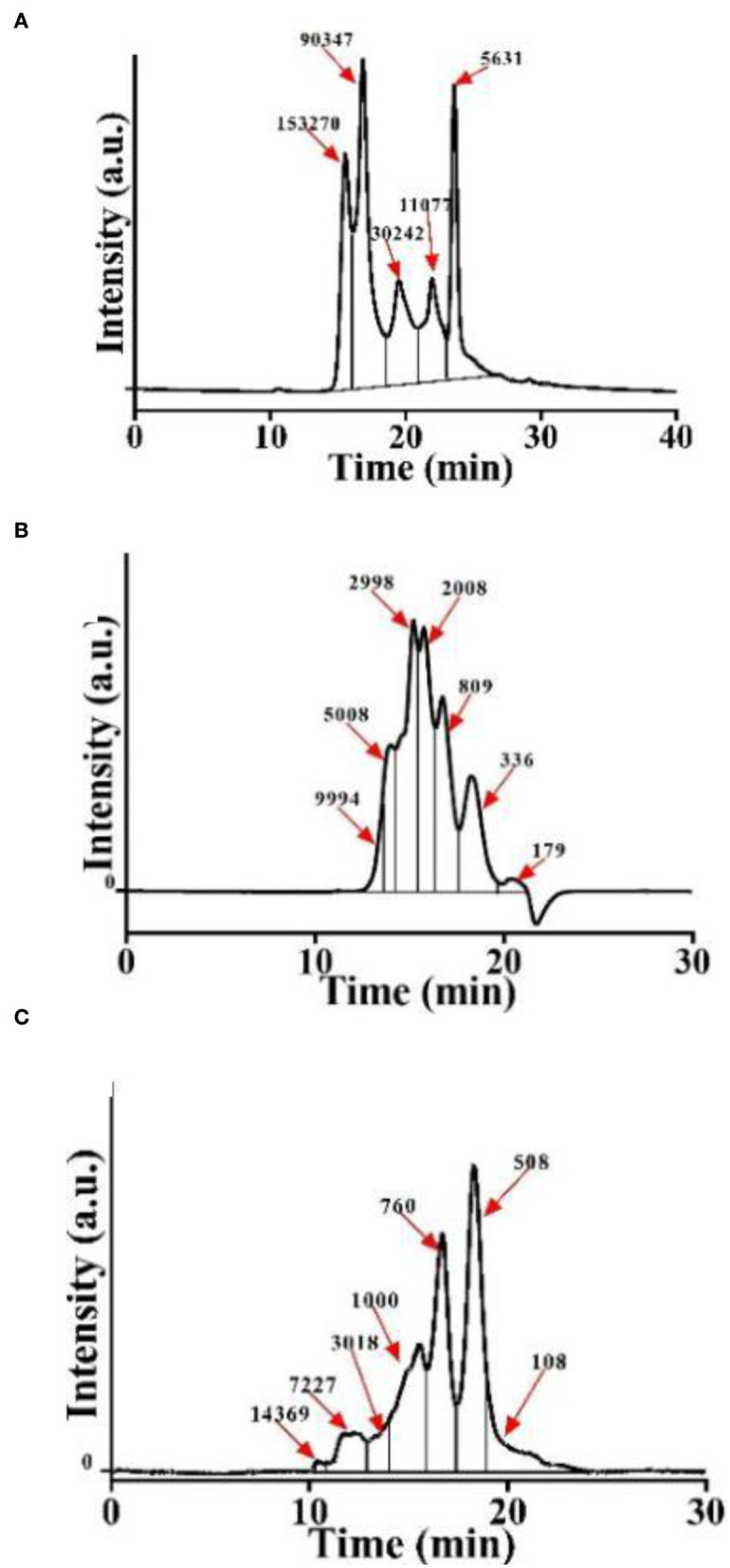
Molecular weight distribution of *CSP*. **(A)** P-1: hydrolyzed by Alcalase–trypsin for 0 min. **(B)** P-2: hydrolyzed by Alcalase–trypsin for 10 min. **(C)** P-3: hydrolyzed by Alcalase–trypsin for 120 min.

Initially, we obtained CSP with different molecular weight distributions by controlling the hydrolysis times of both Alcalase and trypsin. The molecular weight distribution of CSP was analyzed using a dextran gel column (Figure 1). Due to the special network structure of the dextran gel, polypeptides with a large molecular weight flow out from the gel column first, and with the increase in retention time, the molecular weights of the polypeptides decrease. Peak area integration was performed to obtain the polypeptide content in different molecular weight ranges (Table 1). We named the samples P-1, P-2, and P-3 according to the proportions of < 1,000 Da polypeptides. The proportions of < 1,000 Da polypeptides of P-1, P-2, and P-3 were 0.11, 20.26, and 92.72%, respectively. The difference among P-1, P-2, and P-3 was the hydrolysis time; for P-2 and P-3, the hydrolysis time was 20 and 240 min, respectively. As a control, P-1 was C. moschata seed protein without hydrolysis. The proportions of polypeptides < 1,000 Da increased with the increase in hydrolysis time, and the proportion of sample P-3 was the highest (~92.72%).

**Table 1:** Molecular weight distribution of *CSP* (%).

	** < 1,000 Da**	**1–5,000 Da**	**>5,000 Da**
P-1	0.11	17.60	82.29
P-2	20.26	76.38	3.35
P-3	92.72	5.13	2.15

### 3.2. Effects of CSP on cell viability and cell cycle

**Figure 2:**
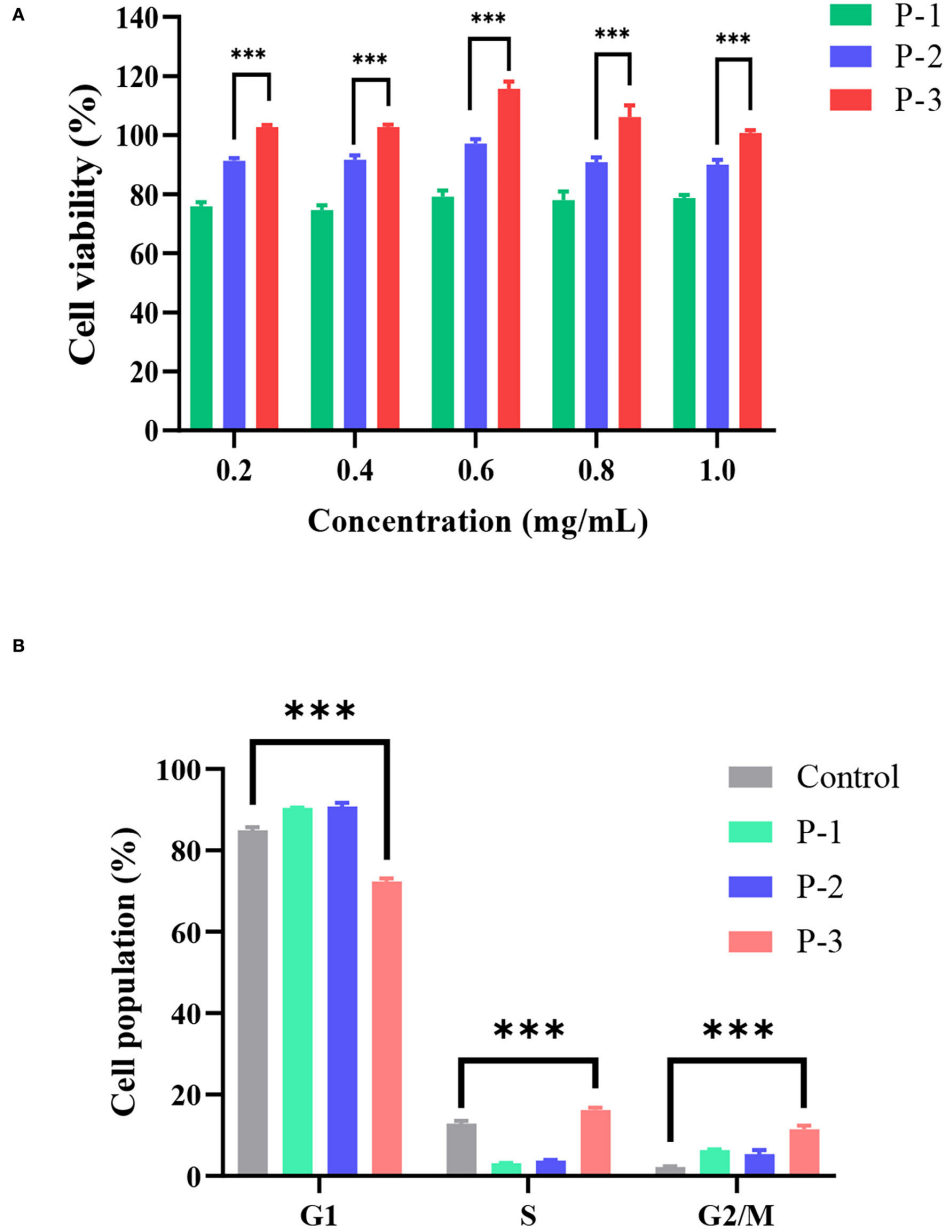
**(A)** Cell viability determined by the MTT assay of HSF cells exposed to 0.2–1.0 mg/ml of P-1, P-2, and P-3 for 24 h. The data were reported as percent viability compared to the control group. **(B)** Cell cycle of HSF cells determined by a flow cytometer exposed to 0.6 mg/ml of P-1, P-2, and P-3 for 24 h. HSF cells only treated with DMEM medium for 24 h were used as the control group. *** indicates differences at a *p*-value of < 0.001.

To investigate the effects of molecular weight distributions of CSP on cell viability, MTT assays were conducted using HSF cell lines. In living cells, MTT can be reduced to water-insoluble formazan and deposited in cells, but dead cells have no such function. Therefore, an MTT kit can evaluate both cell survival rate and cell proliferation ability. As shown in Figure 2A, when the CSP concentration was 0.2–1.0 mg/ml, the cell viability of HSF increased with increasing P-3 proportions. Furthermore, after the HSF cells were treated with 0.2–1.0 mg/ml P-3 and P-2 for 24 h, the cell viability was higher than 100% and slightly < 100%, respectively, indicating that P-3 can but P-2 cannot promote HSF cell proliferation. For P-1 samples, the cell viability was lower than 75%, indicating that P-1 had a certain toxic effect on HSF cells in the concentration range of 0.2–1.0 mg/ml. The higher the proportion of small molecular weight (< 1000 Da) CSPs, the better the ability to promote the cell viability of HSF cells. It also has been reported that the polypeptides produced by the enzymatic hydrolysis method can promote cell growth (30).

Furthermore, the effects of molecular weight distributions of CSP on the cell cycle were investigated. Cell proliferation can be explained by examining the proportion of cells in the G1, S, and G2/M phases. As shown in Figure 2B, compared to the control group, the addition of 0.6 mg/ml P-3 reduced the proportion of cells in the G1 phase from 84.95 to 72.34% and increased the proportion of cells in the S phase from 12.88 to 16.20% and the G2/M phase from 2.18 to 11.46%, indicating that P-3 can promote HSF cell proliferation.

### 3.3. Effects of CSP on the repair of H_2_O_2_-induced oxidatively damaged cells

**Figure 3:**
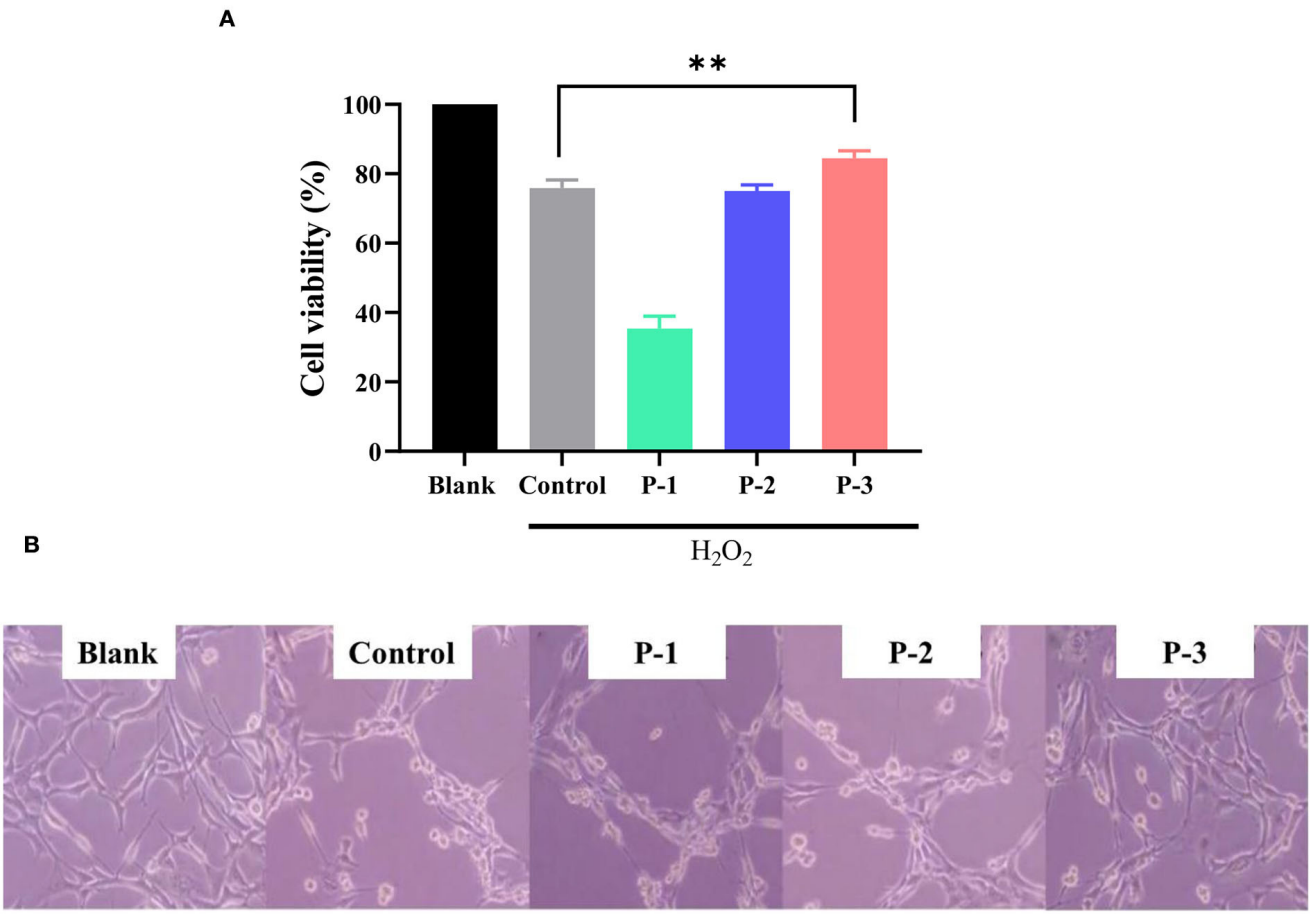
**(A)** Cell viability of the 1.4 mM H_2_O_2_-induced HSF cells after treatment with 0.6 mg/ml of P-1, P-2, and P-3 for 24 h. The data were reported as percent viability compared to the control. **(B)** Morphological observation of H_2_O_2_-induced HSF cells after treatment with 0.6 mg/ml P-1, P-2, and P-3 for 24 h. Control was treated with 1.4 mM H_2_O_2_ for 1 h. HSF cells only treated with DMEM for 24 h were used as the blank. ** indicates the differences at a *p*-value of < 0.05.

Normal cultured cells cannot naturally undergo oxidative damage and enter the senescence period (31). Therefore, oxidative agents were used to induce cellular damage and enter senescence early. The addition of H_2_O_2_ can cause oxidative stress and induce senescence in HSF cells (32). To investigate the repair effects of CSP, H_2_O_2_ was used as an oxidative stress model. As shown in Supplementary Figure S1, the intracellular ROS levels were significantly increased compared to the blank group after 1 h of 1.4 mM H_2_O_2_ treatment, indicating that the oxidative stress-induced premature senescence model was successfully fabricated. As shown in Figure 3A, CSP with different molecular weight distributions showed significantly different repair effects on oxidative stress cells. Cell viability with P-1 was lower compared with the control group. P-2 addition showed no significant effect on the cell viability of H_2_O_2_-induced HSF cells. However, the addition of 0.6 mg/ml P-3 could significantly increase the cell viability by 15.80% compared to the control group. It indicated that P-3 can efficiently prevent the cell death of oxidatively damaged cells.

We observed the cell morphology to intuitively evaluate the effects of CSP with different molecular weight distributions on the repaired oxidatively damaged cells, as shown in Figure 3B. The blank group (without H_2_O_2_) exhibited aggregated and fibrous cells. The number of cells in the culture dish decreased significantly after treatment with 1.4 mM H_2_O_2_ for 1 h. After incubation with 0.6 mg/ml P-1 and P-2 for 24 h, the cell morphology and number did not change significantly compared with the control group (oxidative damage group). However, after the addition of 0.6 mg/ml P-3 for 24 h, the cells became fibrous, and the cell number increased significantly compared with the control group. These observations were consistent with cell viability, which further demonstrated the capability of P-3 in repairing oxidatively damaged HSF cells.

### 3.4. Effects of CSP on the lifespan of H_2_O_2_-induced HSF cells

**Figure 4:**
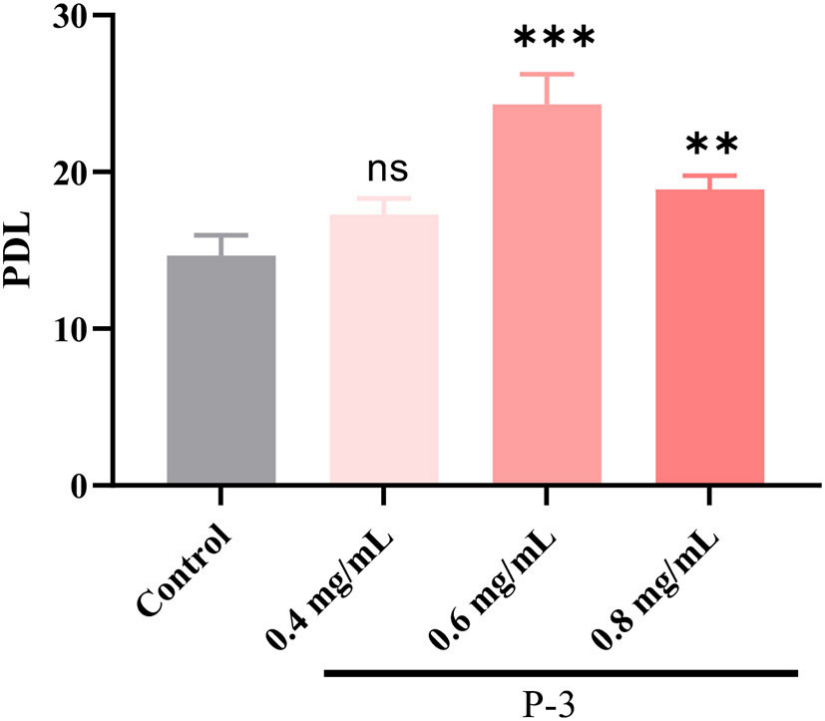
PDL of the 1.4 mM H_2_O_2_-induced HSF cells after treatment with 0.4, 0.6, and 0.8 mg/ml of P-3 for 24 h, respectively. Control was treated with 1.4 mM H_2_O_2_ for 1 h. ** and *** indicate the differences in the *p*-value of < 0.05 and *p*-value of < 0.001, respectively.

According to the aforementioned results, P-3 exhibited an effective repair effect against H_2_O_2_-induced oxidatively damaged cells. Therefore, P-3 was selected for further investigation of its effects on cell lifespan. The PDL can directly reflect the cell lifespan. Hence, the PDL was investigated using the H_2_O_2_-induced oxidatively damaged cells. Since the HSF cell line is more sensitive to H_2_O_2_, it was used in subsequent experiments. The PDLs of HSF cells treated with different concentrations (0.4, 0.6, and 0.8 mg/ml) of P-3 are shown in Figure 4. After treatment with 1.4 mmol/L H_2_O_2_ for 1 h, the PDL value of HSF cells was only 14.85. After adding 0.4, 0.6, and 0.8 mg/ml P-3 to H_2_O_2_-induced HSF cells for 24 h, the PDL values were 17.28, 24.31, and 18.88, respectively. These results suggested that P-3 notably prolonged the mean lifespan of H_2_O_2_-induced HSF cells.

### 3.5 Effects of CSP on H_2_O_2_-induced intracellular ROS level in HSF cells

**Figure 5:**
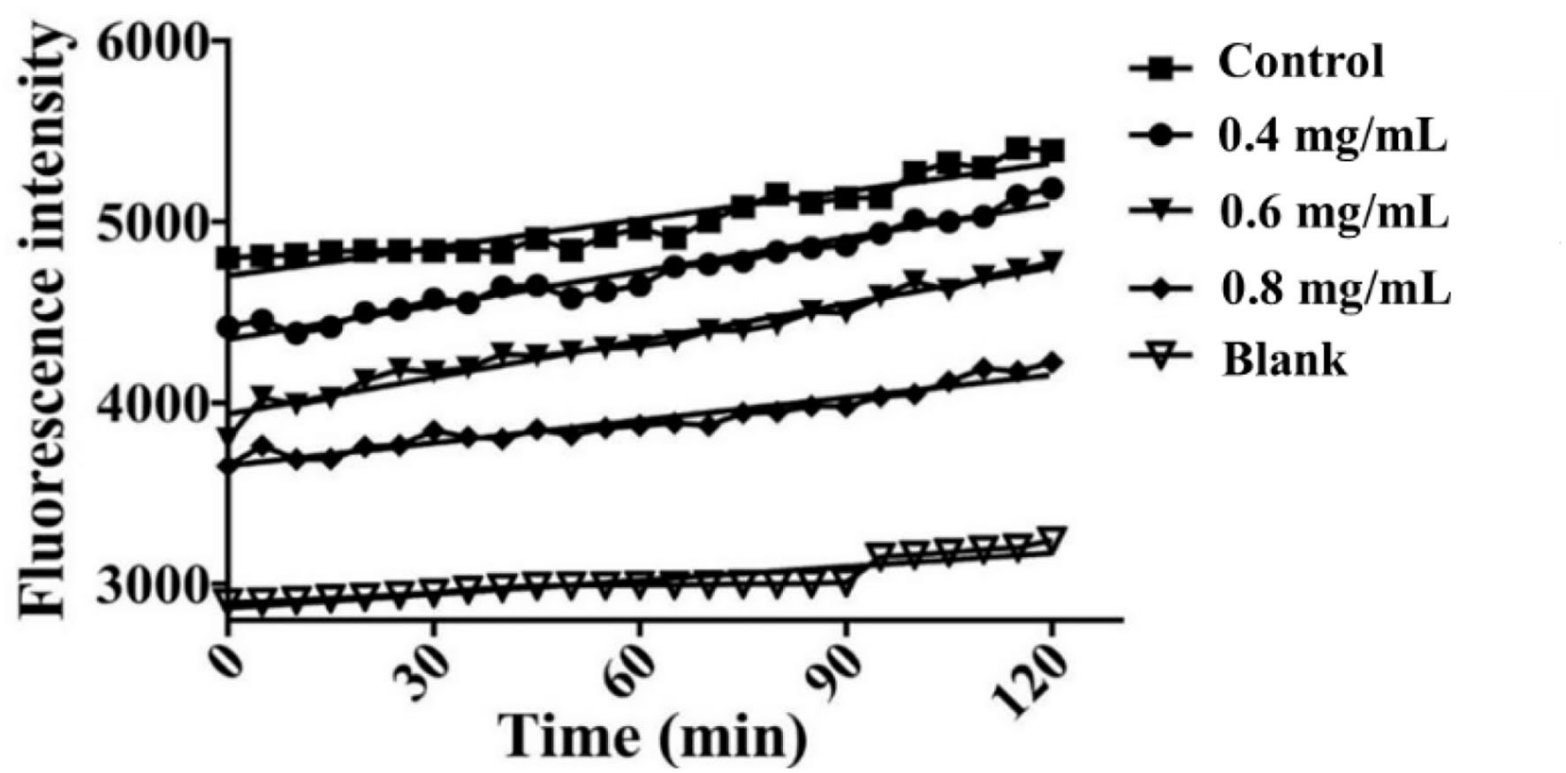
The time-scan of the relative fluorescence intensity for ROS levels of the 1.4 mM H_2_O_2_-induced HSF cells after treatment with 0.4, 0.6, and 0.8 mg/ml P-3 for 24 h, respectively. The HSF cells only treated with DMEM for 24 h were used as blank. Control was treated with 1.4 mM H_2_O_2_ for 1 h.

“Aging free radical theory” states that intracellular ROS plays a vital role in the aging process (33). When there are too many free radicals in the cells, the intracellular antioxidant system cannot remove the excessive free radicals, which results in oxidative damage to the cells and premature aging (34). The ROS levels were measured to further understand the repair effects of *CSP*, as shown in Figure 5. The intracellular ROS level reduction was observed in all HSF cells with 0.4, 0.6, and 0.8 mg/ml P-3. The P-3 addition showed potential ROS scavenging activity and concentration dependence. The low molecular weight (1–3 kDa) peptides derived from sorghum grain have been reported to be effective antioxidants (*in vitro*), as they can scavenge free radicals (35, 36). These findings indicate that the repair activity of P-3 and its inhibition of cell damage mainly depend on its intracellular ROS clearance ability in HSF cells.

### 3.6 Effect of CSP on SOD and GSH levels in H_2_O_2_-induced HSF cells

**Figure 6:**
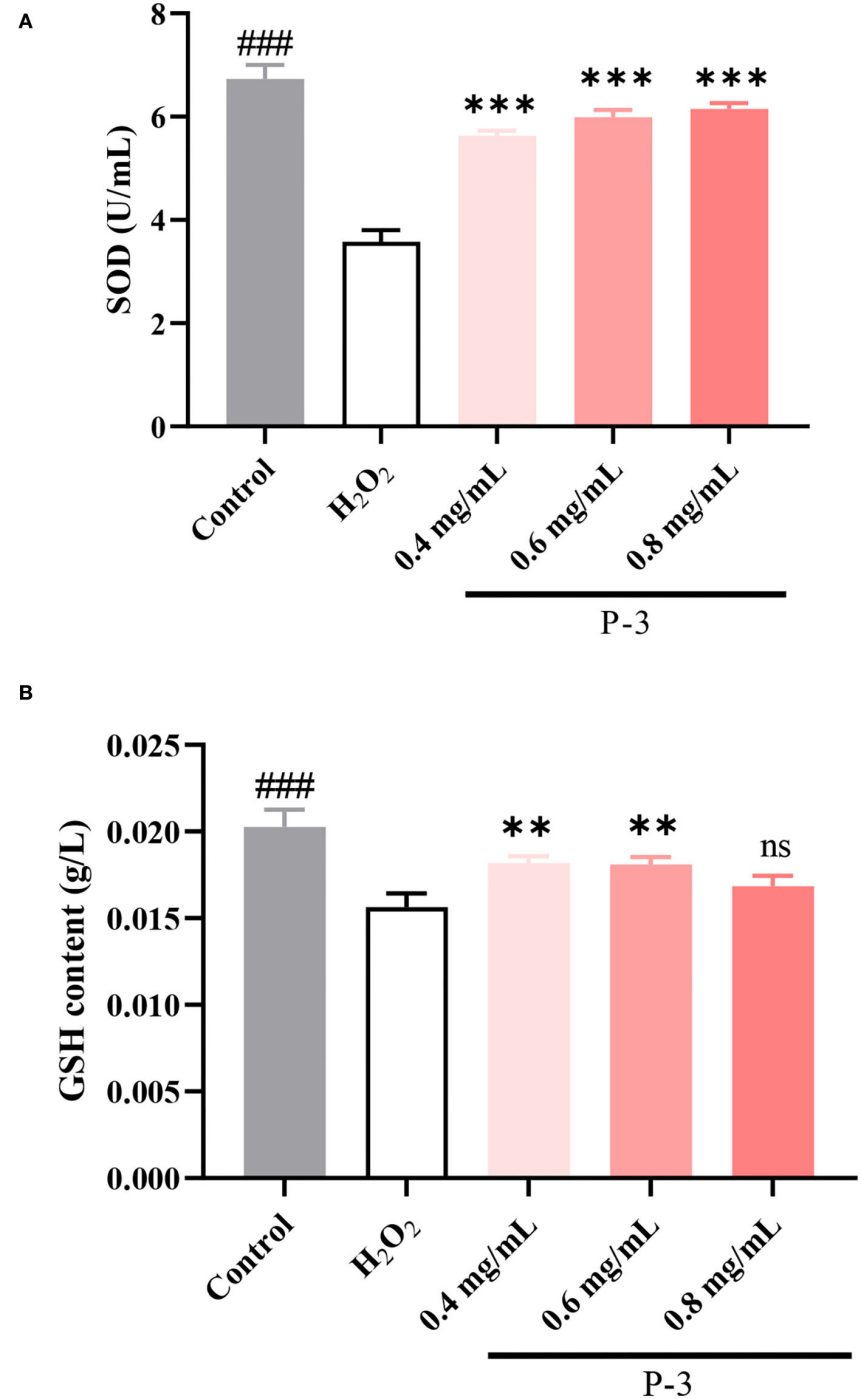
**(A)** SOD activity and **(B)** GSH content of 1.4 mM H_2_O_2_-induced HSF cells after treatment with 0.4, 0.6, and 0.8 mg/ml P-3 for 24 h. The HSF cells only treated by DMEM for 24 h were used as blank. Control was treated with 1.4 mM H_2_O_2_ for 1 h. ** and ***indicate the differences between P-3 and the control at *p*-values of < 0.05 and < 0.001, respectively. ^###^indicates the differences between blank and control at a *p*-value of < 0.001.

Endogenous antioxidants are the products of human metabolism and play an important role in the first line of defense against ROS (37). It includes enzymatic and non-enzymatic antioxidants. Among them, SOD, a kind of enzymatic antioxidant, can remove O_2_-• in cells. When H_2_O_2_ breaks the cell redox balance, the activity of the SOD enzyme would decrease. Another important antioxidant in the cells is the GSH. The main role of GSH in the antioxidant defense system is to directly scavenge free radicals, act as a cofactor for several antioxidant enzymes, and regenerate some antioxidants. As shown in Figure 6A, after the HSF cells were treated with 1.4 mM of H_2_O_2_, the SOD activity was 3.58 U/ml, which was significantly lower than the enzyme activity of the untreated HSF cells (6.73 U/ml). Upon adding 0.4, 0.6, and 0.8 mg/ml P-3, the SOD activity increased to varying degrees and showed a concentration-dependent behavior. The SOD activity was observed to reach up to 6.00 and 6.15 U/ml after adding 0.6 and 0.8 mg/ml P-3, respectively. As shown in Figure 6B, the effects of different concentrations (0.4, 0.6, and 0.8 mg/ml) of P-3 on the GSH content were investigated. The GSH content of HSF cells treated with 1.4 mM H_2_O_2_ was 0.0156 g/L, which was lower than that of 0.0202 g/L of blank (non-damaged) HSF cells. The GSH contents of H_2_O_2_-induced HSF cells with 0.4, 0.6, and 0.8 mg/ml P-3 were 0.0182, 0.0181, and 0.0169 g/L, respectively. The highest GSH content was observed in H_2_O_2_-induced HSF cells with 0.6 mg/ml P-3. These results suggested that the addition of P-3 increased the SOD activity and the GSH content. Thus, the excess active oxygen in HSF cells can be eliminated, thus alleviating oxidative stress and inhibiting premature aging of cells.

### 3.7 Effects of P-3 on premature senescence of H_2_O_2_-induced HSF cells

**Figure 7:**
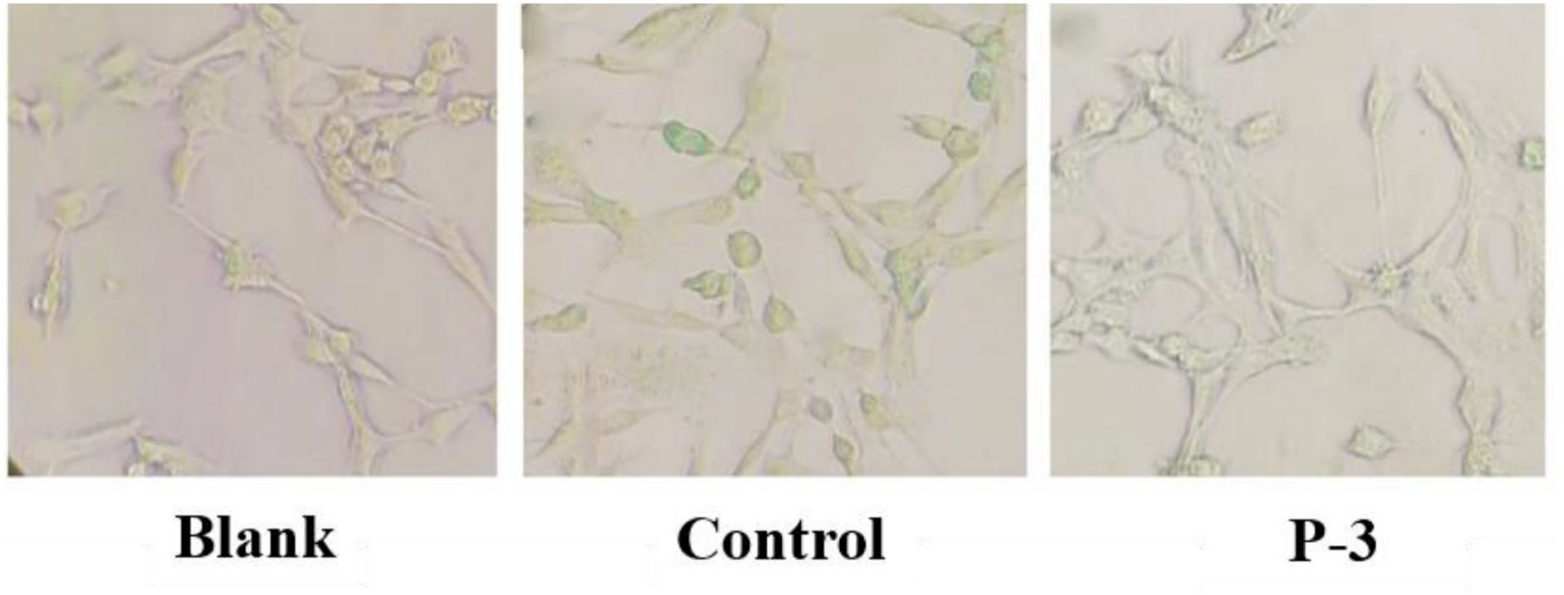
Optical microscope image of cell senescence-associated β-galactosidase staining of 1.4 mM H_2_O_2_-induced HSF cells after treatment with 0.6 mg/ml P-3 for 24 h. The HSF cells only treated by DMEM for 24 h were used as blank. Control was treated with 1.4 mM H_2_O_2_ for 1 h.

Finally, the morphology of H_2_O_2_-induced cells was further observed. The cell senescence-related β-galactosidase staining kit uses X-Gal as a substrate, which can produce a dark blue substance such that the stained senescent cells or tissues can be observed under an ordinary optical microscope. As shown in Figure 7, some cells in the control group were observed to be blue, indicating that the addition of 1.4 mM H_2_O_2_ successfully induced premature cell senescence. With the addition of P-3, the number of blue cells decreased significantly, indicating that the number of senescent cells decreased. Therefore, P-3 can effectively inhibit the premature senescence of HSF cells induced by H_2_O_2_.

## 4. Conclusion

In the present study, *CS* was used as raw materials, and polypeptides with different molecular weight distributions were successfully prepared by controlling the hydrolysis time. Among them, P-3 had the highest proportion of < 1,000 Da molecule polypeptides (92.72%). *CSP* promoted HSF cell growth, and the higher the proportion of small molecular weight *CSP*, the better the cell growth performance. It was found for the first time that *CSP* with a higher proportion of < 1,000 Da polypeptides could effectively inhibit oxidative stress-induced HSF premature senescence and increase the cell lifespan. The SOD enzyme activity and GSH content of HSF cells upon the addition of P-3 were higher than those of the model group. Therefore, the repair effect and premature aging inhibition of P-3 were possibly due to its high ROS scavenging properties by increasing the SOD enzyme activity and GSH content in the redox system of human skin cells. Our results demonstrated that *CSP* with smaller molecular weight polypeptides was a potential and effective antiaging agent *in vitro*, which could be exploited as an antiaging additive to improve skin health.

## Data availability statement

The original contributions presented in the study are included in the article/Supplementary material, further inquiries can be directed to the corresponding authors.

## Author contributions

CL: conceptualization, data curation, and writing—original draft. PW: conceptualization and data curation. CY: supervision and validation. BZ: investigation, writing—review and editing, supervision, and validation. PS: writing—review and editing, supervision, and validation. All authors contributed to the article and approved the submitted version.

## References

[1] Castro-Jácome TP Alcántara-Quintana LE Montalvo-González E Chacón-López A Kalixto-Sánchez MA. del Pilar Rivera M, et al. Skin-protective properties of peptide extracts produced from white sorghum grain kafirins. Indust Crops Prod. (2021) 167:113551. 10.1016/j.indcrop.2021.113551

[2] Dimaki A Kyriazi M Leonis G Sfiniadakis I Papaioannou GT Ioannou E. Diabetic skin and Uv light: protection by antioxidants. Eur J Pharm Sci. (2019) 127:1–8. 10.1016/j.ejps.2018.10.010 30316976

[3] Gu YP Han JX Jiang CP Zhang Y. Biomarkers, oxidative stress, and autophagy in skin aging. Age Res Rev. (2020) 59:1036. 10.1016/j.arr.2020.101036 32105850

[4] Feng S Fu L Wang Y Wang H Yuan M Huang Y. Heat stress resistance effect of flavonoids from penthorum Chinense pursh on caenorhabditis elegans. Pharmacogn Mag. (2019) 15:514–21. 10.4103/pm.pm_500_18

[5] Naeimi AF Alizadeh M. Antioxidant properties of the flavonoid Fisetin: an updated review of *in vivo* and *in vitro* studies. Trends Food Sci Technol. (2017) 70:34–44. 10.1016/j.tifs.2017.10.003

[6] Basu T Panja S Shendge AK Das A Mandal N. A natural antioxidant, tannic acid mitigates iron-overload induced hepatotoxicity in Swiss albino mice through Ros regulation. Environ Toxicol. (2018) 33:603–18. 10.1002/tox.22549 29446234

[7] Taeger J Scherzad A Feineis D Seupel R Hagen R Kleinsasser N. Effects of the novel polyphenol conjugate Dpp-23 on head and neck squamous cell carcinoma cells *in vitro*. Oncol Lett. (2018) 16:654–9. 10.3892/ol.2018.8655 29928453 PMC6006303

[8] Ren Y Wu H Li X Lai F Xiao X. Purification and characterization of high antioxidant peptides from duck egg white protein hydrolysates. Biochem Biophys Res Commun. (2014) 452:888–94. 10.1016/j.bbrc.2014.08.116 25181341

[9] Ochiai A Tanaka S Tanaka T Taniguchi M. Rice bran protein as a potent source of antimelanogenic peptides with tyrosinase inhibitory activity. J Nat Prod. (2016) 79:2545–51. 10.1021/acs.jnatprod.6b00449 27648609

[10] Ookubo N Michiue H Kitamatsu M Kamamura M Nishiki T Ohmori I. The transdermal inhibition of melanogenesis by a cell-membrane-permeable peptide delivery system based on poly-arginine. Biomaterials. (2014) 35:4508–16. 10.1016/j.biomaterials.2014.01.052 24602570

[11] Wei K Guo C Zhu J Wei Y Wu M Huang X. The whitening, moisturizing, anti-aging activities, and skincare evaluation of selenium-enriched mung bean fermentation broth. Front Nutri. (2022) 9:7168. 10.3389/fnut.2022.837168 35369078 PMC8973414

[12] Shahidi F Zhong Y. Bioactive Peptides. J AOAC Int. (2008) 91:914–31. 10.1093/jaoac/91.4.914 18727554

[13] Ceren DD Aysun Y Funda KG Hayrettin D Beraat O. Angiotensin-I-converting enzyme (Ace)-inhibitory peptides from plants. Nutrients. (2017) 9:316–9. 10.3390/nu9040316 28333109 PMC5409655

[14] Albenzio M Santillo A Caroprese M Della MA Marino R. Bioactive peptides in animal food products. Foods. (2017) 6:35–8. 10.3390/foods6050035 28486398 PMC5447911

[15] Fiat AM Migliore-Samour D Jollès P Drouet L Caen J. Biologically active peptides from milk proteins with emphasis on two examples concerning antithrombotic and immunomodulating activities. J Dairy Sci. (1993) 76:301–10. 10.3168/jds.S0022-0302(93)77351-8 8436680

[16] Zimecki M Kruzel ML. Milk-derived proteins and peptides of potential therapeutic and nutritive value. J Exp Ther Oncol. (2007) 6:89–106. 17407968

[17] Majura JJ Cao WH Chen ZQ Htwe KK Li W Du R. The current research status and strategies employed to modify food-derived bioactive peptides. Front Nutri. (2022) 9:50823. 10.3389/fnut.2022.950823 36118740 PMC9479208

[18] Li J Li Y Zhang Y Liu X Zhao Z Zhang J. Toxicity study of isolated polypeptide from wool hydrolysate. Food Chem Toxicol. (2013) 57:338–45. 10.1016/j.fct.2013.03.047 23597444

[19] Ma XM Fan X Wang DP Li XA Wang XY Yang JY. Study on Preparation of Chickpea Peptide and Its Effect on Blood Glucose. Front Nutri. (2022) 9:88628. 10.3389/fnut.2022.988628 36185665 PMC9523602

[20] Li M Zhang Y Xia S Ding X. Finding and isolation of novel peptides with anti-proliferation ability of hepatocellular carcinoma cells from mung bean protein hydrolysates. J Funct Foods. (2019) 62:3557. 10.1016/j.jff.2019.103557

[21] Hu X Liu J Li J Song YQ Chen SJ Zhou SB. Preparation, purification, and identification of novel antioxidant peptides derived from gracilariopsis lemaneiformis protein hydrolysates. Front Nutri. (2022) 9:1419. 10.3389/fnut.2022.971419 35938124 PMC9355161

[22] Chi CF Cao ZH Wang B Hu FY Li ZR Zhang B. Antioxidant and functional properties of collagen hydrolysates from Spanish mackerel skin as influenced by average molecular weight. Molecules. (2014) 19:11211–30. 10.3390/molecules190811211 25090114 PMC6271556

[23] Li Z Wang B Chi C Gong Y Luo H Ding G. Influence of average molecular weight on antioxidant and functional properties of cartilage collagen hydrolysates from Sphyrna Lewini, Dasyatis Akjei, and Raja Porosa. Food Res Int. (2013) 51:283–93. 10.1016/j.foodres.2012.12.031

[24] Dash P Ghosh G. Proteolytic and antioxidant activity of protein fractions of seeds of *Cucurbita moschata*. Food Biosci. (2017) 18:1–8. 10.1016/j.fbio.2016.12.004

[25] Dash P Ghosh G. Amino acid profiling and antimicrobial activity of *Cucurbita moschata* and *Lagenaria Siceraria* seed protein hydrolysates. Nat Prod Res. (2018) 32:2050–3. 10.1080/14786419.2017.1359174 28783965

[26] Teugwa CM Boudjeko T Tchinda BT Mejiato PC Zofou D. Anti-hyperglycaemic globulins from selected Cucurbitaceae seeds used as antidiabetic medicinal plants in Africa. BMC Complement Altern Med. (2013) 13. 10.1186/1472-6882-13-63 23506532 PMC3618205

[27] Fan S Hu Y Li C Liu Y. Optimization of preparation of antioxidative peptides from pumpkin seeds using response surface method. PLoS ONE. (2014) 9:92335. 10.1371/journal.pone.0092335 24637721 PMC3956912

[28] César O Fabiola LGM. Cucurbitaceae seed protein hydrolysates as a potential source of bioactive peptides with functional properties. Biomed Res Int. (2017) 2017:1–16. 10.1155/2017/2121878 29181389 PMC5664370

[29] Wang M Zheng Z Liu C Sun H Liu Y. Investigating the calcium binding characteristics of black bean protein hydrolysate. Food Funct. (2020) 11:8724–34. 10.1039/D0FO01708F 32945323

[30] Chesnokova V Melmed S. Peptide hormone regulation of DNA damage responses. Endocr Rev. (2020) 41:9. 10.1210/endrev/bnaa009 32270196 PMC7279704

[31] Qiu W Chen X Tian Y Wu D Du M Wang S. Protection against oxidative stress and anti-aging effect in drosophila of royal jelly-collagen peptide. Food Chem Toxicol. (2020) 135:110881. 10.1016/j.fct.2019.110881 31622731

[32] Cho EJ Um SI Han JH Kim B Han SB Jeong JH. The cytoprotective effect of rumex aquaticus herba extract against hydrogen peroxide-induced oxidative stress in ags cells. Arch Pharm Res. (2016) 39:1739–47. 10.1007/s12272-016-0863-0 27885462

[33] Han C Someya S. Update on the Free Radical Theory of Aging—The Role of Oxidative Stress in Age-Related Hearing Loss. (2014). 10.1007/978-3-642-30018-9_182

[34] Son TG Kim SJ Kim K Kim MS Chung HY Lee J. Cytoprotective roles of senescence marker protein 30 against intracellular calcium elevation and oxidative stress. Arch Pharm Res. (2008) 31:872–7. 10.1007/s12272-001-1240-3 18704329

[35] Sullivan AC Pangloli P Dia VP. Impact of ultrasonication on the physicochemical properties of sorghum kafirin and *in vitro* pepsin-pancreatin digestibility of sorghum gluten-like flour. Food Chem. (2018) 240:1121–30. 10.1016/j.foodchem.2017.08.046 28946233

[36] Xu S Shen Y Chen G Bean S Li Y. Antioxidant characteristics and identification of peptides from sorghum kafirin hydrolysates. J Food Sci. (2019) 84:2065–76. 10.1111/1750-3841.14704 31313288

[37] Ye HY Li ZY Zheng Y Chen Y Zhou ZH Jin J. The attenuation of chlorogenic acid on oxidative stress for renal injury in streptozotocin-induced diabetic nephropathy rats. Arch Pharm Res. (2016) 39:989–97. 10.1007/s12272-016-0771-3 27289461

