# Supplementary material for: Comparative assessment of Cucurbita moschata seed polypeptides toward the protection of human skin cells against oxidative stress-induced aging
Source: Front Nutr. 2023 Jan 4;9:1091499. doi: 10.3389/fnut.2022.1091499 (PMC9845612; doi:10.3389/fnut.2022.1091499)
Supplement: Supplementary file 1 [file Data_Sheet_1.docx]

**Supporting Information**

Comparative assessment of *Cucurbita moschata* seed polypeptides toward the protection human skin cells against oxidative stress-induced aging

Chunhuan Liu, Peiyu Wang, Cheng Yang, Bingtian Zhao^*^, Peidong Sun ^*^

Key Laboratory of Food Colloids and Biotechnology, Ministry of Education, School of Chemical and Material Engineering, Jiangnan University, Wuxi 214122, P.R. China

Table S1. Preparation conditions of CSP with different molecular weights

| Preparation conditions | P-1 | P-2 | P-3 |
| --- | --- | --- | --- |
| Temperature (℃), pH | 50, 9.0 | 50, 9.0 | 50, 9.0 |
| Alcalase concentration (mg/mL), Time (min) | 0, 0 | 0.025, 10 | 0.025, 120 |
| Temperature (℃), pH | 37, 7.5 | 37, 7.5 | 37, 7.5 |
| Trypsin concentration (mg/mL), Time (min) | 0, 0 | 0.025, 10 | 0.025, 120 |

1. (b)

 **

**Fig. S1**. (a) Cell viability under different concentration of H_2_O_2_; (b) Cell viability under different treating time of H_2_O_2_. Within the testing concentration range, the cell viability showed a concentration dependent behavior after treating with H_2_O_2_ for 1 h. Similarly, the cell viability decreased with the increasing of treating time. HSF cells treated with 1.4 mM H_2_O_2_ for 1 h was chosen as the subsequently oxidative damaging experiment condition.
